# Lethal medical course of an untreated giant cutaneous squamous cell carcinoma

**DOI:** 10.1007/s12024-026-01194-w

**Published:** 2026-02-21

**Authors:** Johanna Hertzberg, Johannes Kleemann, Marcel A. Verhoff, Hannes Gruber

**Affiliations:** 1 Institute of Legal Medicine, University Hospital Frankfurt, Goethe University, Kennedyallee 104, Frankfurt am Main, D-60596 Germany; 2 Institute of Dermatology, University Hospital Frankfurt, Goethe University, Frankfurt am Main, Germany

**Keywords:** Squamous cell carcinoma, Forensic autopsy, Aggressive growth, Thrombosis

## Abstract

Cutaneous squamous cell carcinoma (cSCC) is the second most common malignant skin tumor in Caucasian populations and occurs predominantly on sun-exposed areas of the body. In Germany, which has a nationwide skin cancer screening program, cSCC is usually detected in early tumor stages and can curatively be treated by surgical excision. We report the case of a 59-year-old man who had a giant untreated cSCC of the scalp and died from the local and systemic effects of the aggressively growing tumor. A subsequent medicolegal autopsy was performed. It is noteworthy that the patient had refused medical therapy throughout his lifetime despite the prominence of the tumor. Embarrassment, fear of stigmatization, and denial of the disease itself, as a coping mechanism for self-protection, are possible explanations for giant tumors not being treated, leading to a heightened importance of psycho-oncology in modern cancer therapy to ensure that the mental health of the patient remains intact.

## Introduction

Cutaneous squamous cell carcinoma (cSCC) frequently occurs in sun-exposed areas of the skin, especially on the head and distal extremities [1]. In addition to primary prevention, a national skin cancer screening program was introduced in Germany in 2008, which is covered by the statutory health insurance from the age of 35 years onwards at two-year intervals. [2]. As a result of these widespread preventive measures and the generally good visibility of skin tumors and their precancerous stages, many tumors can be detected early and be successfully treated in their early stages. [3]. The standard therapy, especially for invasive tumors in the head and neck area, consists of micrographic surgery [4]. Radiotherapy is an effective treatment option, for example, in cases of narrow resection margins, lymph node involvement, or other risk constellations [5]. In advanced or metastatic disease, PD-1 checkpoint inhibitors (f.ex. Cemiplimab) have been able to generate high and sustained responses of 40-50% [6, 7].

Despite these numerous diagnostic and therapeutic options and the very good health care availability in Germany, the observation of giant, neglected tumors occurs occasionally. The reasons for tumor denial are complex. Many psychosocial, physiological, and demographic factors have been found to be associated with patients who exhibit tumor denial [8].

## Case report

A 59-year-old man was found incapacitated in his apartment. After a fall in the domestic environment, a giant tumor on the scalp had started to bleed. There was nothing known about the onset and duration of the disease, as the patient did not seek treatment during his lifetime. According to our external anamnestic information, the man had hardly left his apartment since the tumor had become visible. As a result of the fall with the bleeding of the tumor, the man was taken to the hospital. At admission, a CT scan revealed that the giant tumor had already infiltrated and dissolved parts of the skull. No metastases had been detected. The patient died one day after admission to the hospital, since medical treatment could not be performed. After the external postmortem examination, an unclear manner of death was certified. In consequence, the hospital physician informed the police. As a result of the death investigation procedure, a medicolegal autopsy was arranged.

**Fig. 1:**
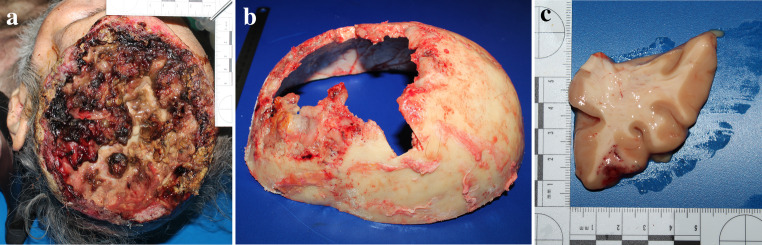
**a**-**c** Macroscopic findings at autopsy in the region of the head and brain. **a** Overview image of the tumor with scale. **b** Parts of the skull bone dissolved by the tumor (condition after specimen collection). **c** Hemorrhage into the brain tissue below the tumor

A forensic postmortem external examination showed a slender body, 170 cm in height, weighing 51 kg (BMI: 17,6—considered significantly underweight). Small incipient decubitus ulcers were observed at the front of the thorax, on the knees, on the back of the left foot, and on the back of several toes, indicating a prolonged prone position. Based on the circumstances of discovering the man in his apartment and the visible pressure sores, it was assumed that the patient was lying at least partially on their front after a fall. A tumor, shown in Fig. 1, measuring 16.5: 14 cm and covering almost the entire scalp, was found, which had disintegrated the skullcap in an area measuring 9: 7 cm.

**Fig. 2:**
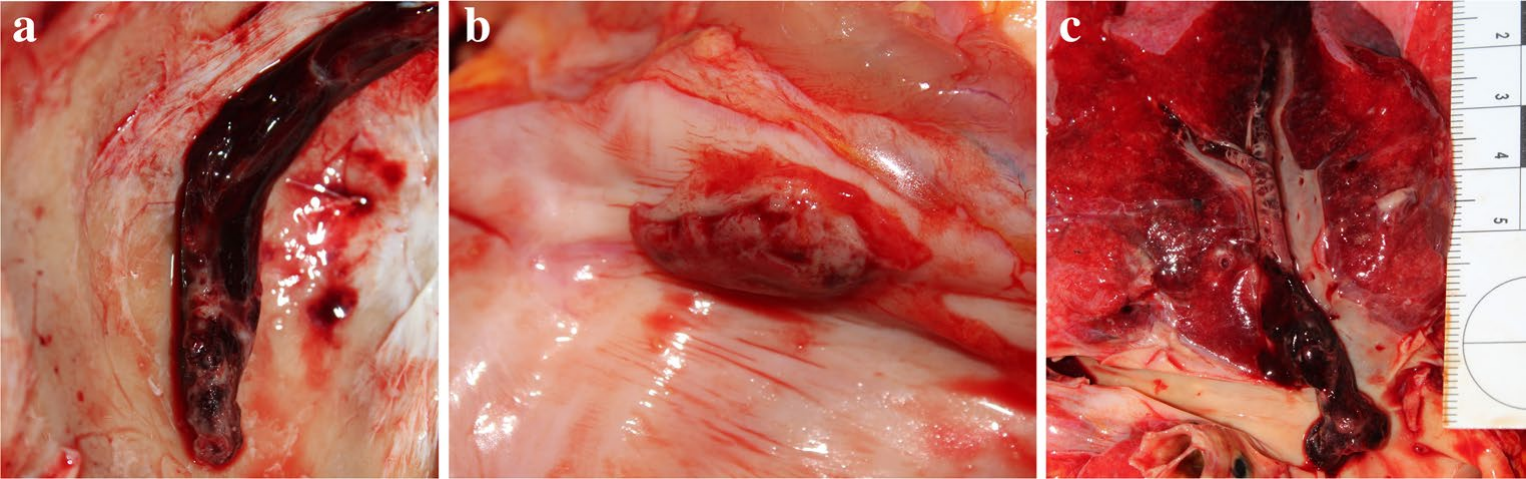
**a**-**c** Overview of thromboembolic events found during postmortem examination. **a** Thrombosis of the left sigmoid sinus. **b** Mural, already organized thrombus of the left brachiocephalic vein. **c** Thrombus in the pulmonary arteries

Below, a hemorrhage area of up to 1.5 cm in size was visible on the cerebral cortex. In addition, an already organized thrombus was found in the left brachiocephalic vein, shown in Fig. 2, with retrograde formations of blood clots extending through the left internal jugular vein up into the left sigmoid sinus. Furthermore, beginning to form, but not yet adhering to the wall, pulmonary embolism, deep vein thrombosis in the lower legs, and inflammatory changes of the lung were evident. The right heart was dilated, and the fatty liver showed signs of acute congestion. The left adrenal gland presented with a small hemorrhage. Kidneys and intestines showed no major pathology. In conclusion of these findings, death can be attributed to the tumor condition. The leading cause of death is thought to be pulmonary embolism, although sinus vein thrombosis would also be a plausible explanation for the death. A combination of the two findings would also be a plausible explanation.

**Fig. 3:**
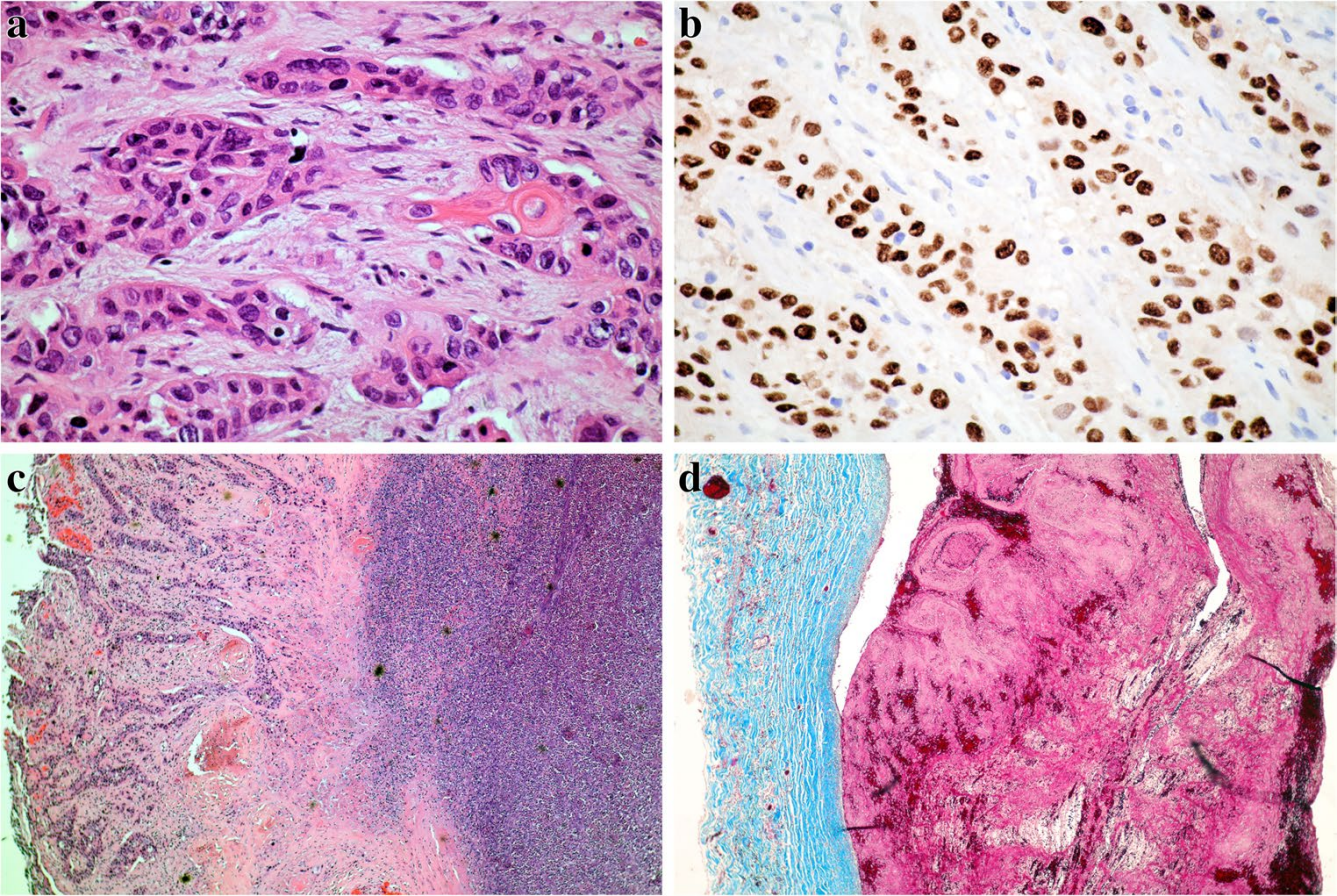
**a**-**d** Histological findings. **a** Moderately differentiated squamous cell carcinoma of the skin with single horny pearls (HE 40x). **b** Positive nuclear stainability of tumor cells for p 63 (Immunhistochemistry p 63 40 x). **c** Tumor with reference to the hard meninges with inflammatory infiltration below (HE 4x). d Already organized, wall-bound thrombus in the area of the brachiocephalic vein (Masson Goldner 4x)

The histological examination, shown in Fig. 3, revealed a moderately differentiated (G2), partially keratinizing squamous cell carcinoma. cSCC arises from the malignant degeneration of keratinocytes. This tumor had a locally aggressive growth with dissolution of the cranial dome and growth into the pachymeninx. Inflammatory infiltration was seen underneath the dura mater. Immunohistochemistry revealed an expression pattern typical of squamous cell carcinoma with marked nucleolar positivity for p63. Despite the locally aggressive growth, no metastases could be detected.

## Discussion/Conclusion

Due to incomplete recording of non-melanoma skin cancer in the German cancer registry, it is difficult to state the exact mortality rates of cSCC [9]. However, several studies indicate that both incidence and mortality for cSCC are increasing in Germany [10, 11]. Findings of giant cSCC with drastic local and systemic complications, as presented in this case, are rare in Germany. Finding explanations for these occurrences can be challenging. A possible explanation for the growth of a prominent skin cancer to giant sizes without the patient seeking out medical treatment might be embarrassment or fear of social stigmatization, as has been observed with other prominent diseases [12, 13] and might even have medicolegal implications after death, for example, by reducing the quality of the postmortem examination [14]. Other factors may include the relatively slow, largely painless progression of the disease, possibly coupled with denial of the disease itself, as a coping mechanism for self-protection [15]. It is interesting to note that, in many cases, it is a sudden change in the lesion, such as hemorrhage, that leads the patients to seek medical attention, as in the presented case [8]. Another explanation for the growth might be the stressful situation that accompanies the affliction, causing mental illness to develop or an existing mental illness to exacerbate [16]. In modern cancer therapy, this has led to psycho-oncology becoming increasingly important in the holistic treatment of patients to ensure that not only the cancer itself is cured, but also that the mental health of the patient remains intact [17]. However, an influence of the corona pandemic is also conceivable. Initial statistical analyses and surveys indicate that many patients did not attend medical appointments, especially preventive appointments, in fear of contagion during the pandemic, which may lead to a higher occurrence of such pronounced findings [18].

## Data Availability

All data generated or analyzed during this study are included in this article. Further inquiries can be directed to the corresponding author.
